# Worst case optimization for interfractional motion mitigation in carbon ion therapy of pancreatic cancer

**DOI:** 10.1186/s13014-016-0705-8

**Published:** 2016-10-07

**Authors:** Julian Steitz, Patrick Naumann, Silke Ulrich, Matthias F. Haefner, Florian Sterzing, Uwe Oelfke, Mark Bangert

**Affiliations:** 1 German Cancer Reserach Center - DKFZ, Im Neuenheimer Feld 280, Heidelberg, Germany; 2 Department of Radiation Oncology, University Hospital Heidelberg, Heidelberg, Germany; 3 Joint Department of Physics at The Institute of Cancer Research and The Royal Marsden NHS Foundation Trust, London, UK

**Keywords:** Carbon ion therapy, Treatment planning, Worst case optimization, Pancreatic cancer, Interfractional motion

## Abstract

**Introduction:**

The efficacy of radiation therapy treatments for pancreatic cancer is compromised by abdominal motion which limits the spatial accuracy for dose delivery - especially for particles. In this work we investigate the potential of worst case optimization for interfractional offline motion mitigation in carbon ion treatments of pancreatic cancer.

**Methods:**

We implement a worst case optimization algorithm that explicitly models the relative biological effectiveness of carbon ions during inverse planning. We perform a comparative treatment planning study for seven pancreatic cancer patients. Treatment plans that have been generated using worst case optimization are compared against (1) conventional intensity-modulated carbon ion therapy, (2) single field uniform dose carbon ion therapy, and (3) an ideal yet impractical scenario relying on daily re-planning. The dosimetric quality and robustness of the resulting treatment plans is evaluated using reconstructions of the daily delivered dose distributions on fractional control CTs.

**Results:**

Idealized daily re-planning consistently gives the best dosimetric results with regard to both target coverage and organ at risk sparing. The absolute reduction of *D*_95_ within the gross tumor volume during fractional dose reconstruction is most pronounced for conventional intensity-modulated carbon ion therapy. Single field uniform dose optimization exhibits no substantial reduction for six of seven patients and values for *D*_95_ for worst case optimization fall in between. The treated volume (*D*>95 *%* prescription dose) outside of the gross tumor volume is reduced by a factor of two by worst case optimization compared to conventional optimization and single field uniform dose optimization. Single field uniform dose optimization comes at an increased radiation exposure of normal tissues, e.g. ≈2 Gy (RBE) in the mean dose in the kidneys compared to conventional and worst case optimization and ≈4 Gy (RBE) in *D*_1_ in the spinal cord compared to worst case optimization.

**Conclusion:**

Interfractional motion substantially deteriorates dose distributions for carbon ion treatments of pancreatic cancer patients. Single field uniform dose optimization mitigates the negative influence of motion on target coverage at an increased radiation exposure of normal tissue. Worst case optimization enables an exploration of the trade-off between robust target coverage and organ at risk sparing during inverse treatment planning beyond margin concepts.

## Introduction

Even though the incidence of pancreatic cancer remains rather low with around twelve cases per 100,000 [1], pancreatic cancer is the fourth leading cause of cancer death in developed countries with two- and five-year survival rates between 40–50 % and 5–20 %, respectively [2, 3]. The standard of care in pancreatic cancer is surgical resection of the primary tumor. This approach, however, is often impossible due to vessel involvement making definitive or neoadjuvant therapies indispensable to achieve tumor control or tumor shrinkage before a potential resection. Besides systemic therapies, radiation therapy plays an important role in this context.

The effectiveness of irradiation for locally advanced pancreatic cancer is limited by the tolerance doses of adjacent normal tissues. Even though the development of intensity-modulated radiation therapy provided considerable improvements regarding dose conformity and enabled the application of integrated boost concepts, further dose escalation is desired to enhance efficacy. First studies in this regard with carbon ion therapy [4], that might also exhibit a benefit in relative biological effectiveness (RBE) [5], showed promising clinical results [6].

In pancreatic cancer treatments, the high spatial accuracy of radiation therapy is contrasted by anatomical variations due to varying organ filling, tissue shrinkage or expansion, and respiratory motion [7]. These motion phenomena are especially critical for the application of particle therapy [8, 9]. However, sophisticated online motion compensation strategies as currently exercised for photons [10, 11] remain impractical for particle therapy in today’s clinical practice due to hardware limitations.

In this work we investigate the potential of worst case optimization for interfractional offline motion mitigation in treatments of pancreatic cancer. So far, worst case optimization [12–14] has only been applied for photon and proton therapy with a focus on range and setup uncertainties. It has been shown that worst case optimization can minimize the sensitivity to range or setup uncertainties and reduces the normal tissue dose compared to conventional margin concepts [15–17]. Li et al. [18] investigated the role of worst case optimization with regard to anatomical changes for lung cancer patients treated with proton beams. They concluded that the dose variations due to anatomy changes can be reduced yet still re-planning might be necessary in some cases.

Here, we present the first implementation of worst case optimization [12] in combination with an effect-based model to account for the RBE of carbon ions [19]. Furthermore we investigate the potential to use worst case optimization based on range and setup uncertainties to compensate for motion and deformation phenomena. We evaluate the robustness of the treatment plans that have been generated on recontoured fractional control CTs, i.e., an independent test set. The results are interpreted in comparison to conventional intensity-modulated carbon ion therapy, single field uniform dose carbon ion therapy, and an ideal yet impractical scenario relying on daily re-planning, i.e., adaptive intensity-modulated carbon ion therapy.

## Methods

We performed a comparative treatment planning study for seven pancreatic cancer cases. Imaging data and corresponding segmentations of the patient cohort are explained in “Patient characteristics”, the considered treatment planning strategies are explained in “Treatment planning strategies”, and the metrics used for comparison are explained in “Comparison criteria”. All treatment planning approaches facilitate an optimization strategy that is directly based on the biological effect to account for the RBE of carbon ions, as explained in “Effect-based inverse planning”.

### Patient characteristics

The patient cohort consists of seven pancreatic cancer patients with one planning CT and three to four fractional control CTs at the same spatial resolution and image quality (30 data sets in total). Fractional control CTs were rigidly registered to the planning CT by matching the position of the spinal column.

For all patients, the gross tumor volume (GTV) was segmented by physicians on all CTs. According to [20], the planning target volume (PTV) was directly generated through uniform expansion of the GTV by 15 mm. In agreement with the National Comprehensive Cancer Network guidelines, the prescribed dose was 54 Gy (RBE) in 27 fractions. Besides target volumes, also kidneys, spinal cord, liver, stomach, and intestine were segmented by physicians on all 30 CTs.

### Effect-based inverse planning

Clinical treatment planning with carbon ions requires an explicit modeling of the RBE on top of the physically absorbed dose. Therefore, we perform inverse planning for all treatment planning strategies based on an objective function *F* that directly depends on the biological effect *ε* [19]

1 $${} F(\varepsilon)=\sum\limits_{v\in Targets} \left({p_{v}^{U}} {F_{v}^{U}} (\varepsilon) + {p_{v}^{O}} {F_{v}^{O}} (\varepsilon)\right) + \sum\limits_{v\in OAR} {p_{v}^{O}} {F_{v}^{O}} (\varepsilon).   $$

The superscripts *U* and *O* denote under-effect and over-effect; $p_{v}^{U/O}$ is the penalty factor for volume *v* for under- and over effect. The components of the objective function are defined as

2 $$ {F_{v}^{O}}(\varepsilon)=\sum\limits_{i\in v} \left[ \varepsilon_{i} - {\varepsilon_{v}^{O}} \right]_{+}^{2}  $$

3 $$ {F_{v}^{U}}(\varepsilon)=\sum\limits_{i\in v} \left[ {\varepsilon_{v}^{U}} - \varepsilon_{i} \right]_{+}^{2}  $$

where ${\varepsilon _{v}^{O}}$/${\varepsilon _{v}^{U}}$ are thresholds for the desired maximum/minimum effect in *v* and *ε*_*i*_ denotes the actual effect in voxel *i*. The positivity operator ensures that only violated thresholds contribute to the objective function, i.e., [*x*]_+_=*x* for *x*>0 and [*x*]_+_=0 otherwise.

The effect $\varepsilon _{i} = \alpha _{i} d_{i} + \beta _{i} {d_{i}^{2}}$ depends not only on the dose *d*_*i*_ in voxel *i* but also on the radiation sensitivity parameters of the linear quadratic model (LQM) *α*_*i*_ and *β*_*i*_. As different carbon ion pencil beams with different radiation qualities superimpose in voxel *i*, the optimization facilitates a synergistic effect [21] according to

4 $$ \begin{aligned} \varepsilon_{i}(\mathbf{w}) &= \alpha_{i} (\mathbf{w}) d_{i}(\mathbf{w}) + \beta_{i}(\mathbf{w}){d_{i}^{2}}(\mathbf{w})\\ &= \sum\limits_{j=1}^{N} \alpha_{ij}D_{ij}w_{j} + \left(\sum\limits_{j=1}^{N} \sqrt{\beta_{ij}}D_{ij}w_{j} \right)^{2} \end{aligned}  $$

where the matrices *D*_*ij*_ and *α*_*ij*_/*β*_*ij*_ specify the dose contribution from pencil beam *j* to voxel *i* and the LQM parameters of pencil beam *j* in voxel *i*. *w*_*j*_ denotes the weight of pencil beam *j*; consequently the dose in voxel *i* is given by $d_{i} = \sum _{j} D_{ij} w_{j}$. For our simulations we assume *β*_*ij*_ to be constant [19].

The effect-based optimization is performed with our in-house treatment planning software KonRad [22]. *α*_*ij*_ matrices are computed based on tabulated data stemming from simulations of the local effect model I [23] assuming a constant *α*/*β*=2 for all tissues for technical reasons. The carbon ion dose calculation relies on a pencil beam algorithm facilitating a double Gaussian parameterization in lateral direction to accurately account for the low dose halo beyond the Bragg peak [24]. Due to the large lateral low dose extend of the individual pencil beams, we implemented a dose-dependent voxel sampling strategy following [25] to reduce the memory requirements to store the *D*_*ij*_ and *α*_*ij*_ matrices.

### Treatment planning strategies

Our study compares four treatment planning strategies

**Fig. 1:**
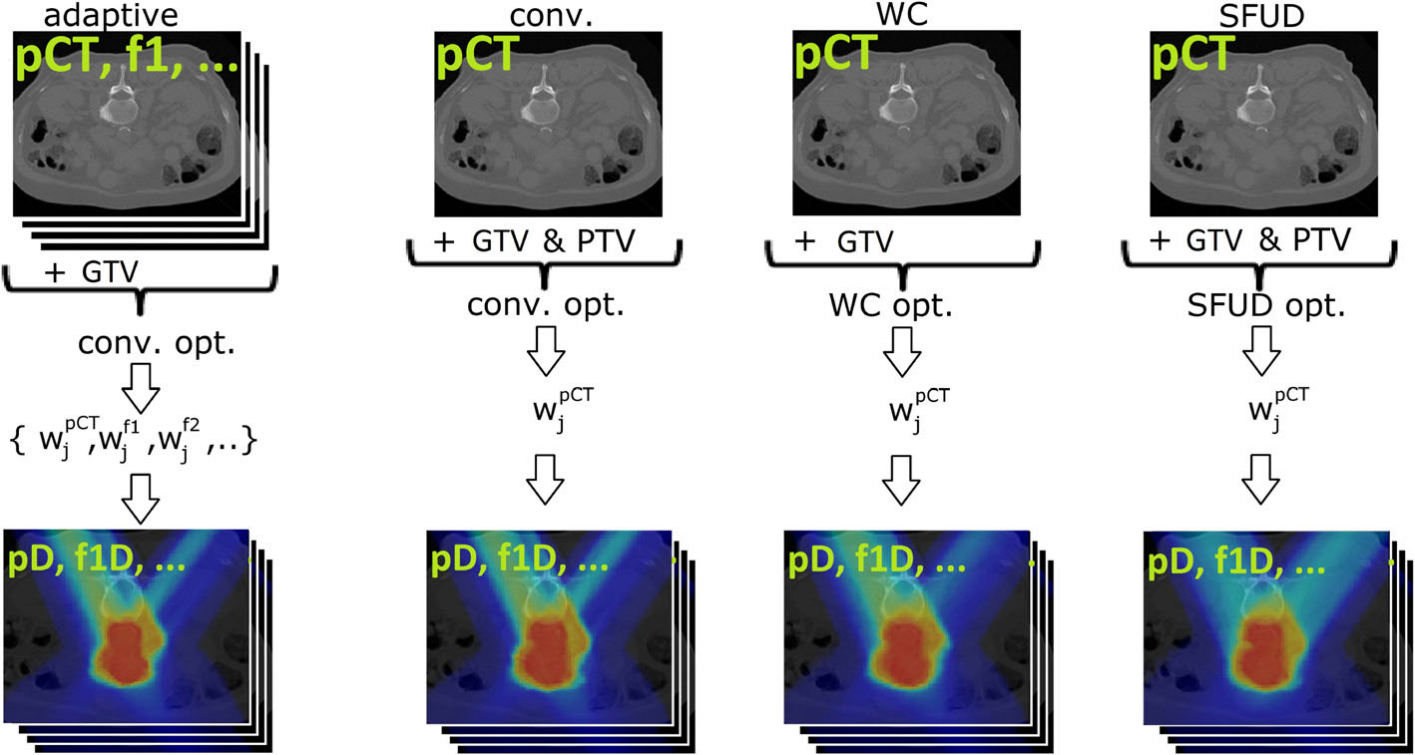
Treatment planning study. Schematics of the four treatment planning approaches. While the conventional, worst case, and SFUD approaches only perform one optimization on the planning CT (pCT), the adaptive approach facilitates a re-optimization on every fractional CT (f1,…). Consequently, we only have one set of pencil beam weights $w_{j}^{pCT}$ for the conventional, worst case, and SFUD approach that are used to compute the fractionally delivered dose on every CT. For the adaptive approach, we have multiple sets of pencil beam weights $\{w_{j}^{pCT},w_{j}^{f1},\dots \}$ - one for each CT

**Fig. 2:**
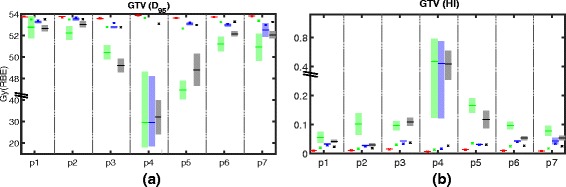
**a** *D*_95_ and (**b**) target homogeneity index *HI* within the GTV and for adaptive (*red*), conventional (*green*), SFUD (*blue*), and worst case (*black*) optimization for all patients (p1 – p7). The planning scenario corresponds to the cross (*x*), the mean of all dose recalculations on fractional control CTs corresponds to the minus sign (–), and the shaded area around the mean indicates ± one standard deviation. Observe the interrupted y axis

1. Conventional intensity-modulated carbon ion therapy.
2. Single field uniform dose (SFUD) carbon ion therapy.
3. Worst case optimized intensity-modulated carbon ion therapy.
4. Adaptive intensity-modulated carbon ion therapy.

which are explained in detail in “Conventional intensity-modulated carbon ion therapy” to “Adaptive intensity-modulated carbon ion therapy”; a schematic overview is given in Fig. 1. All generated treatment plans apply two dorsal fields aiming for the gap between the spinal cord and the kidneys corresponding to gantry angles around 330° and 30°. To avoid range uncertainties emerging from a partial irradiation through the patient couch, we assume the patient to be in prone position. The lateral spot distance was 4 mm. The penalties and maximum/minimum dose input parameters for the objective function (1) can be found in Table 1. Note that the penalty factors for target volumes were carefully adjusted individually for every treatment planning method (but kept the same for different patients) to achieve similar target coverage in the planning scenario for conventional intensity-modulated carbon ion therapy, SFUD carbon ion therapy, and worst case optimization (compare Fig. 2).

**Table 1:** Objective function parameters for all volumes *v*

*v*	*d* ^*O*^ [Gy(RBE)]	*d* ^*U*^ [Gy(RBE)]	*p*_*v*_
GTV	54	54	Individual
Spinal Cord	10	–	200
Kidneys	5	–	250
Stomach	15	–	15
Intestine	15	–	15
Liver	15	–	15
Normal Tissue	15	–	5

*d* ^*O*/*U*^ hresholds for over- and under-dose, respectively. Note that the dose values have to be converted into thresholds in over- and under-effect according to the LQM for effect-based optimization. For the target volumes, the penalties *p*_*v*_ were adjusted individually for all four treatment planning strategies but kept the same for OARs

#### Conventional intensity-modulated carbon ion therapy

For conventional intensity-modulated carbon ion therapy we optimize a treatment plan based on the planning CT for every patient. Potential uncertainties are accounted for by creating a PTV with a uniform margin as detailed in “Patient characteristics”. The optimizer has the full flexibility to distribute the dose over the individual beams. This may result in a pronounced modulation of the two individual fields that only together yield a homogeneous RBE weighted dose within the target volume.

#### Single field uniform dose carbon ion therapy

For SFUD^1^ carbon ion therapy we optimize a treatment plan based on the planning CT for every patient. Potential uncertainties are accounted for by creating a PTV with a uniform margin as detailed in “Patient characteristics”. The optimizer is restricted to construct a homogeneous RBE weighted dose within the target volume with fields that - taken alone - also yield a homogeneous RBE-weighted dose in the target volume ([26], Chapter 8.C). Due to the explicit RBE dependence, we have to apply the LQM to adjust the prescription doses for the individual beams to achieve the desired target dose as detailed in Appendix A.

#### Worst case optimized intensity-modulated carbon ion therapy

Worst case optimization uses a modified objective function

5 $$ \tilde{F}(\varepsilon(\mathbf{w}))=F(\varepsilon_{nom}(\mathbf{w})) + p_{wc} \cdot F(\varepsilon_{wc}(\mathbf{w}))  $$

during inverse planning [12]. Besides the nominal effect *ε*_*nom*_ also a worst case effect *ε*_*wc*_ is considered within an additional term in the objective function. *p*_*wc*_ denotes the relative weighting of the worst case effect; we set *p*_*wc*_=1 [12]. Six worst case scenarios corresponding to lateral shifts of the carbon ion beams and over- and underestimation of the carbon ion range within the patient are computed. The worst case effect *ε*_*wc*_ is constructed in every iteration of the optimization through voxelwise combination of the minimum effect in the target volumes and the maximum effect in the OARs observed in the nominal treatment scenario or in one of the six worst case scenarios [12, 27]. Since each voxel is considered independently, the worst case effect distribution is nonphysical, but it serves as a lower bound of the plan quality [12]. Worst case optimization does not rely on PTV margins. Worst case optimization only considers the GTV definition; the high dose region that guarantees adequate target coverage is automatically determined by the optimizer according to a priori specified range and setup uncertainties. We assume 15 mm setup and 15 mm range uncertainties for our simulations, which corresponds to the PTV margin used for conventional and SFUD planning (compare “Patient characteristics”).

#### Adaptive intensity-modulated carbon ion therapy

For adaptive intensity-modulated carbon ion therapy, we optimize a treatment plan before every treatment fraction based on up-to-date imaging data, i.e., the fractional control CTs. We assume perfect knowledge about the patient anatomy and plan directly on the GTV without safety margin. This approach is clinically unrealistic but serves as a valuable upper bound regarding dosimetric treatment quality in our study.

### Comparison criteria

The quality of the individual treatment plans is analyzed based on dose volume histogram (DVH) statistics of the dose calculations on the planning and fractional control CTs. We use *D*_95_ to quantify target coverage. Dose delivered to OARs is evaluated based on the mean dose for the kidneys, liver, stomach and intestine and *D*_1_ for the spinal cord, liver, stomach and intestine. *V*_95_ is used as a measure for the treated volume outside of the GTV and consequently target conformity, *V*_107_ is used as a measure for the volume of hot spots inside the GTV, and the homogeneity index *H* *I*=(*D*_5_−*D*_95_)/*D*_*mean*_ is used as a measure of dose homogeneity within the GTV. Statistical significance (*p*<0.05) is tested with a paired, two-sided Wilcoxon signed rank test.

## Results

Treatment plans for all planning approaches were optimized and recalculated with our in-house research treatment planning software KonRad [22]. The voxel resolution was 2.62×2.62×2.62 mm^3^ resulting in dose influence and *α* matrices comprising up to 120 GB. The run time for inverse planning including dose calculation was up to 20 h for the worst case optimization and 2 hours for the conventional optimization. Both the dose calculation and optimization algorithm facilitate a single-threaded implementation that has not been optimized for speed.

**Fig. 3:**
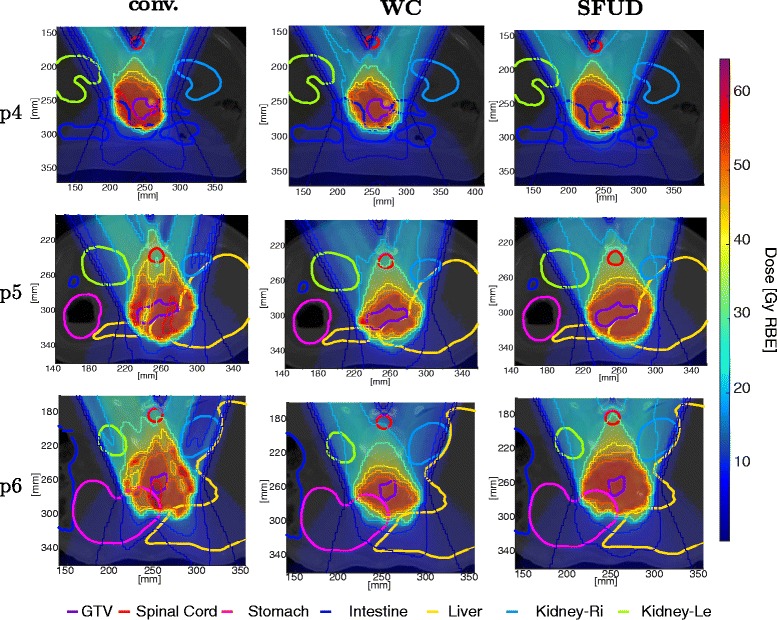
Example dose distributions for three tested optimization methods for different patients. The *left column* shows the conventional optimization which shows pronounced hot/cold spots. The *mid column* shows the worst case optimization, which generates a smaller high dose region around the GTV without pronounced hot/cold spots. The *right column* shows the SFUD optimization also without pronounced hot/cold spots

Figure 2 a shows the stability of the dose distribution within the GTV over multiple fractions. The prescribed dose was 54 Gy (RBE) delivered over 27 fractions. Using adaptive re-planning, it is possible to guarantee an adequate target coverage over all fractions for all patients. All other treatment planning approaches yield a clear decline in *D*_95_ in the fractionally reconstructed dose distributions as compared to the original planning scenario. For conventional IMPT optimization, the average *D*_95_ minus one standard deviation ($\bar {D}_{95}-\sigma $) drops below 51.3 Gy (RBE), i.e., 95 % of the prescribed dose, for five patients. For worst case optimization, $\bar {D}_{95}-\sigma $ drops below 51.3 Gy (RBE) for three patients. For SFUD optimization, $\bar {D}_{95}-\sigma $ drops below 51.3 Gy (RBE) for one patient. The absolute reduction in *D*_95_ is most pronounced for conventional IMPT (except patient p3); SFUD exhibits no substantial reduction in *D*_95_ for six of seven patients. Values for *D*_95_ for worst case optimization fall between conventional and SFUD optimization (except patient p3). The observed differences in *D*_95_ between conventional IMPT and worst case optimization, between conventional IMPT and SFUD, and between worst case optimization and SFUD are statistically significant (*p*=5.8·10^−5^, *p*=5.8·10^−5^, and *p*=8.3·10^−4^, respectively). Target homogeneity is visualized in Fig. 2 b. While there is barely a difference between the planning scenario and the fractionally reconstructed dose in target homogeneity index *HI* for adaptive re-planning, the biggest detoriation is observed for conventional IMPT. For SFUD, we observe a moderate increase in *HI*; values for worst case optimization usually fall between conventional IMPT and SFUD (except patient p3). The spatial pattern of the underdosage within the GTV is shown in Fig. 3. While the cold spots within the GTV occur at the edges for worst case and SFUD optimization, we also see cold spots appearing in the middle of the GTV for conventional IMPT optimization.

Note that it was not possible to achieve adequate target coverage using offline techniques only for patient p4. Both an SFUD concept using conventional margins and the worst case optimization fail as the observed motion greatly exceeds the considered margins and uncertainties in caudal cranial direction.

**Fig. 4:**
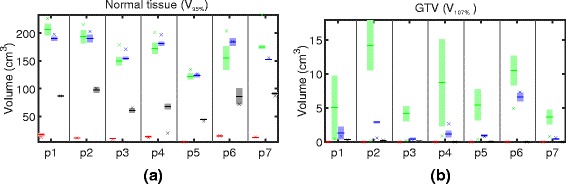
**a** High dose volumes above 95 % outside of the GTV and (**b**) high dose volume above 107 % inside the GTV for adaptive (*red*), conventional (*green*), SFUD (*blue*), and worst case (*black*) optimization for all patients (p1 – p7). The planning scenario corresponds to the cross (*x*), the mean of all dose recalculations on fractional control CTs corresponds to the minus sign (–), and the shaded area around the mean indicates ± one standard deviation

The treated volume with D≥51.3 Gy (RBE), i.e. 95 % of the prescribed dose, outside of the GTV is depicted in Fig. 4 a. Here, we see a very consistent behavior: Even though worst case optimization facilitates the same assumptions about the underlying uncertainty as the margin based approaches, it yields a reduction of the high dose volume around the GTV of about 50 % (which roughly corresponds to about 100 cm^3^) compared to SFUD and conventional optimization. This can also be seen on the transversal dose distributions in Fig. 3. The observed differences in the high dose volume outside of the GTV between conventional IMPT and worst case optimization and between SFUD and worst case optimization are statistically significant (*p*=1.7·10^−6^ and *p*=1.7·10^−6^); the differences between conventional IMPT and SFUD are not statistically significant (*p*=0.090). The risk of undesired hot spots can be reduced using worst case optimization as depicted in Fig. 4 b. Note that the numbers reflect fractional recalculations. Considering an accumulated dose over the entire treatment, these effects might be moderated by averaging effects.

**Fig. 5:**
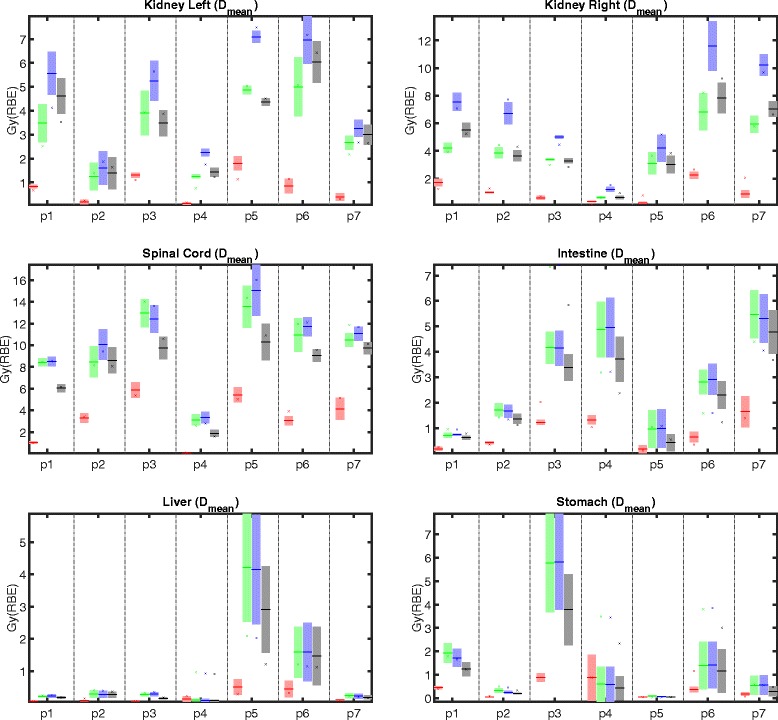
Mean dose in different OARs for adaptive (*red*), conventional (*green*), SFUD (*blue*), and worst case (*black*) optimization for all patients (p1 – p7). The planning scenario corresponds to the cross (*x*), the mean of all dose recalculations on fractional control CTs corresponds to the minus sign (–), and the shaded area around the mean indicates ± one standard deviation

**Fig. 6:**
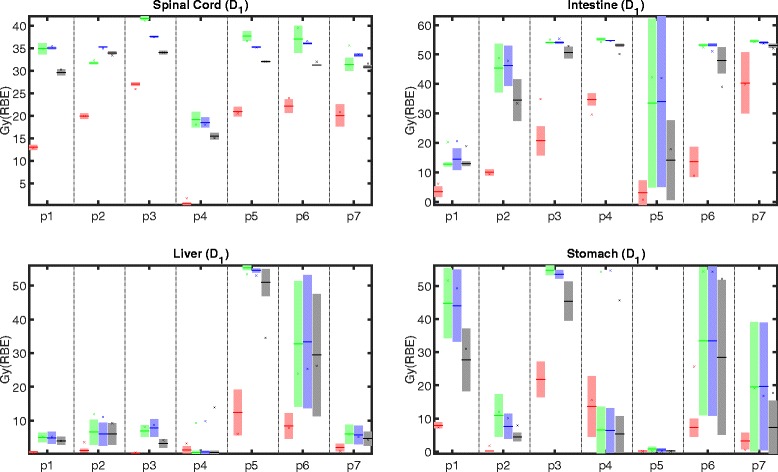
*D*_1_ in different OARs for adaptive (*red*), conventional (*green*), SFUD (*blue*), and worst case (*black*) optimization for all patients (p1 – p7). The planning scenario corresponds to the cross (*x*), the mean of all dose recalculations on fractional control CTs corresponds to the minus sign (–), and the shaded area around the mean indicates ± one standard deviation

Figures 5 and 6 depict mean and maximum dose statistics for selected OARs. As expected, the idealized adaptive treatment planning approach yields the best sparing of OARs in the planning scenarios as well as the best stability of the dose statistics over multiple fractions. For the other treatment planning strategies, we see that conventional IMPT and worst case optimization usually yield superior sparing of OARs compared to SFUD optimization. This can be explained by the additional constraint that every individual field has to deliver a homogeneous target dose for SFUD. Hence, it is not possible to use intensity-modulation to down-regulate parts of one beam and up-regulate parts of another field to spare OARs at constant target coverage.

This issue especially compromises sparing of the kidneys for SFUD optimization, as depicted in Fig. 5. For the majority of patients, the additional freedom in intensity-modulation for conventional IMPT and worst case optimization enables a reduction of more than 2 Gy (RBE) in the mean doses in the kidneys when compared to SFUD optimization. The observed mean dose differences for both the left and right kidney between conventional IMPT and worst case optimization, between conventional IMPT and SFUD, and between worst case optimization and SFUD are statistically significant (Left kidney: *p*=0.0243, *p*=1.7·10^−6^, and *p*=1.7·10^−6^, respectively. Right kidney: *p*=0.017, *p*=1.7·10^−6^, and *p*=1.7·10^−6^, respectively.).

The maximum dose in the spinal cord resulting from worst case optimization is lower than the maximum dose resulting from conventional and SFUD optimization for all patients except patient p2. This applies to both the planned dose distribution as well as the fractionally reconstructed doses. Due to the lack of a fixed PTV margin, worst case optimization may adjust the high dose region around the target to better spare this OAR in the beam path. The observed differences in *D*_1_ in the spinal cord between conventional IMPT and worst case optimization as well as between between worst case optimization and SFUD are statistically significant (*p*=5.3·10^−5^ and *p*=1.7·10^−6^, respectively); differences between conventional IMPT and SFUD are not statistically significant (*p*=0.544).

For liver, stomach, and intestine, worst case optimization consistently yields lower mean dose and *D*_1_ for both the planning scenario and the fractionally reconstructed dose compared to conventional IMPT and SFUD. This behavior can again be explained by the additional freedom of the optimizer to balance an expansion of the high dose region around the GTV against OAR sparing. However, these observations should be interpreted with care as liver, stomach, and intestine are given substantially lower weight during the optimization (compare Table 1).

## Discussion

This paper presents the first implementation of effect-based worst case optimization for carbon ion therapy. The clinical potential of this optimization strategy is evaluated in a treatment planning study focusing on pancreatic cancer patients. To benchmark worst case optimization, our study also considers treatment plans that were generated by conventional intensity-modulated carbon ion therapy optimization and SFUD optimization based on the planning CT. The robustness of the resulting treatment plans is evaluated by dose recalculations on fractional control CTs. Hence, we provide an evaluation of competing offline motion compensation strategies on an independent test set with anatomical motion stemming from different treatment days. Furthermore, our results are interpreted in the context of an idealized treatment planning approach that facilitates daily re-planning on fractional control CTs.

With worst case optimization becoming a commercially available treatment planning option [28], our paper addresses the clinical need for an independent evaluation of the underlying algorithmic concepts. We make an important contribution to the understanding of worst optimization for clinical treatment planning and highlight the implications of inverse planning without a PTV margin.

Overall we observe a substantial improvement in robustness of target coverage for treatment plans facilitating worst case optimization compared to treatment plans facilitating conventional optimization. SFUD optimization is the most robust approach with regard to target coverage (leaving the technically infeasible approach of daily re-planning without PTV margin aside) but it comes at an increased radiation exposure of normal tissues. This has to be attributed to the additional condition that the optimizer cannot modulate the intensity of individual fields to spare OARs in the beam path, e.g. the kidneys as depicted in Fig. 3. The increased exposure of normal tissues for conventional and SFUD optimization is also caused by the fixed PTV margin around the target. While these optimization techniques inevitably fill the PTV margin with the prescription dose, worst case optimization has the possibility to trade any potential expansion of the high dose region beyond the target volume off against sparing of adjacent normal tissues. Using objective function (1) in combination with the parameters detailed in Table 1, this resulted in a reduction of the volume irradiated at doses above 95 % of the prescribed dose of about 100 cm^3^ in our treatment planning study. We want to point out that a different choice of optimization parameters would yield not only a different volume irradiated at more than 95 % of the prescription dose but also a different trade-off with regard to treatment plan robustness. Just like conventional PTV margin concepts have been carefully validated for photons [29], a widespread clinical application of worst case optimization demands a thorough understanding of the influence of objective function parameters on the trade-off in treatment plan robustness. While it is feasible to develop site-specific robustness recipies for worst case optimization [30], it remains unclear how a generalized parameter recipe for worst case optimization could look like making further research - also considering alternative approaches [31, 32] - necessary.

Our work is in agreement with previous studies demonstrating that range and setup uncertainties can be used within worst case optimization to make treatment plans more robust against anatomical variations [33]. Furthermore, we reconfirm that offline motion compensation alone does not yield the one ideal treatment plan that can be applied over all treatment fractions. Spontaneous motion phenomena in the order of cm as regularly occurring within the abdomen make daily re-planning highly desirable in clinical practice [33].

Technically, our study only considers interfractional motion. However, as both the planning CT and the fractional control CTs stem from different intrafractional motion phases, the results of our study may also generalize towards motion during the treatment.

Our study reports dose statistics from the recalculated fraction doses. We refrain from interfractional dose accumulation to rule out potential uncertainties emerging from deformable image registration which is subject to pronounced uncertainty given the low contrast in the soft tissue within the abdomen [34]. While it is possible to identify anatomical structures on the control CTs for experienced clinicians, automated image processing requires higher contrast, which could be provided in the future through the application of daily MR guidance [35].

**Fig. 7:**
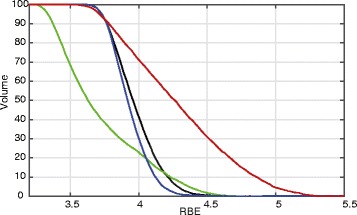
RBE volume histogram for patient p6 within the GTV. Worst case (*black*) and SFUD (*blue*) optimization yield more homogeneous RBE distributions within the target volume compared to the conventional (*green*) and adaptive optimization (*red*)

The presented algorithm is the first robust optimization method that explicitly takes the three-dimensional RBE of carbon ions into account. As such it yields a homogeneous RBE-weighted dose distribution within the target. This is also achieved by applying a more homogeneous RBE distribution within the target as depicted in Fig. 7. While RBE values for conventional RBE optimization range from 3.3 to 4.6, RBEs for worst case optimization range from 3.7 to 4.4 which is comparable to SFUD optimization. Note that adaptive re-planning generally yields higher RBE values within the target. Due to the absence of margins, high LET values from the target edges move into the GTV, making margin reductions additionally attractive for carbon ion therapy.

## Conclusion

Our study confirmed that interfractional motion substantially deteriorates dose distributions for carbon ion treatments of pancreatic cancer patients. Single field uniform dose optimization proved to be an adequate means to mitigate the negative influence of interfractional motion on target coverage. However, the increased robustness was associated with an increased radiation exposure of normal tissues when compared to conventional IMPT optimization.

Furthermore we presented and evaluated the first implementation of effect-based worst case optimization for carbon ion therapy. Within our treatment planning study, we showed that worst case optimization can be a suited tool to balance treatment plan robustness, target coverage, and sparing of normal tissues. In contrast to conventional optimization and single field uniform dose optimization, which both rely on a fixed margin concept, worst case optimization can freely adjust the high dose region around the target during inverse planning. This enables an exploration of the trade-off between robust target coverage and organ at risk sparing during inverse treatment planning. For carbon ion treatments for pancreatic cancer that resulted in improved robustness of target coverage at reduced normal tissue dose compared to conventional optimization. Compared to single field uniform dose optimization, worst case optimization enabled substantial reductions of normal tissue dose at a moderate decrease in robust target coverage.

Idealized considerations with regard to daily adaptation of a carbon ion treatment plan, however, demonstrated that worst case optimization based on the planning CT does not suffice to deliver the best possible dose distribution for every patient on every treatment day.

## Endnote

^1^ SFUD optimization is also often called “single beam optimization”.

## Appendix A

## Prescription for SFUD optimization

Effect-based SFUD optimization requires a non-linear adjustment of the dose to be applied by the individual fields. According to the LQM, the effect for a fractionated radiation treatment is given by

6 $$ E = n \left(\alpha d + \beta d^{2} \right) = \alpha D + \beta D d  $$

with the fraction dose *d*, the total dose *D*, and the number of fractions *n*. Considering two different fractionation schemes *a* and *b* with the same number of fractions and identical effect, we have

7 $$ \frac{D_{a}}{D_{b}} = \frac{d_{b} + \alpha/\beta}{d_{a} + \alpha/\beta}.  $$

Postulating that *D*_*b*_ is applied in *n*·*η* equal fractions (with the number of fields *η*) and that *D*_*a*_ is applied in *n*·1 equal fractions (i.e., with a single field), we obtain

8 $$ \frac{d_{a}}{\eta d_{b}} = \frac{d_{b} + \alpha/\beta}{d_{a} + \alpha/\beta}  $$

which can be rearrangened to yield the dose to be applied by an individual field of an SFUD treatment with *η* beams

9 $$ d_{b} = -\frac{\alpha}{2\beta} \pm \sqrt{\frac{\alpha^{2}}{4\beta^{2}} + \frac{d_{a}}{\eta}\left(d_{a} + \frac{\alpha}{\beta} \right)}.  $$

The solution considering the minus sign can be discarded as it would yield an unphysical negative dose *d*_*b*_. In combination, the *η* fields will yield approximately a homogeneous RBE-weighted dose *d*_*a*_ in the target volume for one fraction.

## Abbreviations

conv. (opt.): Conventional (optimization)
DVH: Dose volume histogram
GTV: Gross tumor volume
HI: Homogeneity index
IMPT: Intensity modulated particle therapy
LET: Linear energy transfer
LQM: Linear quadratic model
OAR: Organ at risk
PTV: Planning target volume
RBE: Relative biological effectiveness
SFUD: Single field uniform dose
WC (opt.): Worst case (optimization)
